# Association between low-grade inflammation and Breast cancer and B-cell Myeloma and Non-Hodgkin Lymphoma: findings from two prospective cohorts

**DOI:** 10.1038/s41598-018-29041-1

**Published:** 2018-07-17

**Authors:** Eloise Berger, Cyrille Delpierre, Fatemeh Saberi Hosnijeh, Michelle Kelly-Irving, Lutzen Portengen, Ingvar A. Bergdahl, Ann-Sofie Johansson, Vittorio Krogh, Domenico Palli, Salvatore Panico, Carlotta Sacerdote, Rosario Tumino, Soterios A. Kyrtopoulos, Paolo Vineis, Marc Chadeau-Hyam, Roel Vermeulen, Raphaële Castagné, EnviroGenoMarkers, Beatrice Melin, Per Lenner, Benedetta Bendinelli, Maria Botsivali, Aristotelis Chatziioannou, Ioannis Valavanis, Barbara Bodinier, Javiera Garrido-Manriquez, Toby J. Athersuch, Benoît Liquet, Henk Lokhorst, Panagiotis Georgiadis, Jos C. S. Kleinjans, Theo M. C. M. de Kok, Hector C. Keun, Rachel Kelly, Goran Hallmans, Euripides G. Stephanou, Antonis Myridakis, Manolis Kogevinas, Lucia Fazzo, Marco De Santis, Pietro Comba, Hannu Kiviranta, Panu Rantakokko, Riikka Airaksinen, Paivi Ruokojarvi, Mark Gilthorpe, Sarah Fleming, Thomas Fleming, Yu-Kang Tu, Thomas Lundh, Kuo-Liong Chien, Wei J. Chen, Wen-Chung Lee, Chuhsing Kate Hsiao, Po-Hsiu Kuo, Hung Hung, Shu-Fen Liao

**Affiliations:** 1 LEASP, UMR 1027, Inserm-Université Toulouse III Paul Sabatier, Toulouse, France; 2 Institute for Risk Assessment Sciences, Division of Environmental Epidemiology, Utrecht University, Utrecht, The Netherlands; 3 Immunology Department, Erasmus University Medical Center, Rotterdam, The Netherlands; 4 Department of Biobank Research, Umeå University, Umeå, Sweden; 5 Department of Radiation Sciences, Oncology, Umeå University, Umeå, Sweden; 6 Fondazione IRCCS- Instituto Nazionale dei Tumori, Milan, Italy; 7 Istituto per lo Studio e la Prevenzione Oncologica (ISPO Toscana), Florence, Italy; 8 Department of Clinical Medicine and Surgery, University of Naples Federico II, Naples, Italy; 9 Piedmont Reference Centre for Epidemiology and Cancer Prevention (CPO Piemonte), Turin, Italy; 10 Cancer registry and Histopathology Unit, Azienda Ospedaliera ‘Civile –M.P.Arezzo’, Ragusa, Italy; 11 National Hellenic Research Foundation, Institute of Biology, Medicinal Chemistry and Biotechnology, Athens, Greece; 12 HuGeF, Human Genetics Foundation, Torino, Italy; 13 Department of Epidemiology and Biostatistics, School of Public Health, Imperial College London, London, UK; 14 MRC-PHE Centre for Environment and Health, Imperial College, London, London, UK; 15 Division of Computational and Systems Medicine, Department of Surgery and Cancer, Faculty of Medicine, Imperial College London, London, UK; 16 Laboratoire de Mathématiques et de leurs Applications, Université de Pau et des Pays de l’Adour, UMR CNRS 5142 Pau, France; 17 ARC Centre of Excellence for Mathematical and Statistical Frontiers, Queensland University of Technology (QUT), Brisbane, Australia; 18 Department of Hematology, University Medical Center Utrecht, Utrecht, The Netherlands; 19 National Hellenic Research Foundation, Institute of Biology, Pharmaceutical Chemistry and Biotechnology, Athens, Greece; 20 Department of Toxicogenomics, Maastricht University, Maastricht, The Netherlands; 21 Division of Cancer, Department of Surgery and Cancer, Imperial College London, Institute of Reproductive and Developmental Biology (IRDB), Hammersmith Hospital, London, UK; 22 Channing Division of Network Medicine, Department of Medicine, Brigham and Women’s Hospital and Harvard Medical School, Boston, USA; 23 Nutrition Research, Department of Public Health and Clinical Medicine, and Department of Biobank Research, Umeå University, Umeå, Sweden; 24 Environmental Chemical Processes Laboratory, University of Crete, Heraklion, Greece; 25 ISGlobal, Centre for Research in Environmental Epidemiology (CREAL), Barcelona, Spain; 26 Istituto Superiore di Sanita, Rome, Italy; 27 National Institute for Health and Welfare, Kuopio, Finland; 28 University of Leeds, Leeds, United Kingdom; 29 Lund University, Lund, Sweden; 30 National Taiwan University, Taipei, Taiwan

## Abstract

Chronic inflammation may be involved in cancer development and progression. Using 28 inflammatory-related proteins collected from prospective blood samples from two case-control studies nested in the Italian component of the European Prospective Investigation into Cancer and nutrition (n = 261) and in the Northern Sweden Health and Disease Study (n = 402), we tested the hypothesis that an inflammatory score is associated with breast cancer (BC) and Β-cell Non-Hodgkin Lymphoma (B-cell NHL, including 68 multiple myeloma cases) onset. We modelled the relationship between this inflammatory score and the two cancers studied: (BC and B-cell NHL) using generalised linear models, and assessed, through adjustments the role of behaviours and lifestyle factors. Analyses were performed by cancer types pooling both populations, and stratified by cohorts, and time to diagnosis. Our results suggested a lower inflammatory score in B-cell NHL cases (β = −1.28, p = 0.012), and, to lesser, extent with BC (β = −0.96, p = 0.33) compared to controls, mainly driven by cancer cases diagnosed less than 6 years after enrolment. These associations were not affected by subsequent adjustments for potential intermediate confounders, notably behaviours. Sensitivity analyses indicated that our findings were not affected by the way the inflammatory score was calculated. These observations call for further studies involving larger populations, larger variety of cancer types and repeated measures of larger panel of inflammatory markers.

## Introduction

Inflammation is a key pathway in many diseases including cancer^1^, a leading cause of death worldwide^2^. More specifically inflammation is one of the enabling hallmarks of cancer^3^ and is thought to influence several phases of cancer development including initiation and progression^4^ through both intrinsic (genetic events) and extrinsic (soluble mediators) pathways^5^. Epidemiological, pharmacological and genetic research provide solid evidence that inflammation can increase cancer risk and promote tumour progression^6,7^. Epidemiological studies have already examined the link between inflammation and risk of some cancers including bladder, oesophageal, ovarian, prostate or thyroid cancer^6^ but also colorectal cancer^8^ and others^7^.

While breast cancer (BC) is the most commonly diagnosed cancer among women worldwide, B-cell lymphomas (BCLs) are the most common hematopoietic cancers in both men and women in the developed world. Few epidemiological studies have investigated the association between blood levels of cytokines^9,10^, CRP^11–13^ and/or immune related factor^9^ and risk of BC in prospective settings and results were inconsistent^9–15^. Several prospective studies have also examined the association between B-cell Non-Hodgkin Lymphoma (B-cell NHL) and circulating levels of inflammatory markers providing strong evidence of a subtle role of inflammation in B cell NHL development and progression^16–26^. These studies have suggested an increased risk of B cell NHL and/or its subtypes with increased blood levels of interleukin^17–20,23–25^, chemokines^16,25^, immune activation markers^16,17,22^ and growth factor^19^. While other studies reported negative association of interleukin with risk of B cell NHL^18,23^.

The prevalence of these two cancers and the conflicting results regarding the role of inflammation underline that BC and B-cell NHL are particularly relevant to explore the role of inflammation in cancer initiation.

Previous studies generally focus on the concentration levels of individual biomarkers, and since these biomarkers are systemic, associations between individual biomarkers and cancer may not be always apparent. The use of lower resolution summary scores may improve statistical power and reveal associations with cancer risk^27,28^. In the present work, we investigate inflammatory scores to ascertain their link with cancers onset. We used data from the EnviroGenoMarkers (EGM) project designed as two nested case-control studies including breast cancer (BC) and Β-cell Non-Hodgkin Lymphoma (B-cell NHL, including multiple myeloma) cases within two cohorts. Specifically, we constructed our inflammatory score using a large panel of cytokines, chemokines and growth factors measured in prospectively collected peripheral blood samples in 268 participants from the Italian component of the European Prospective Investigation into Cancer and Nutrition (EPIC-Italy) and 492 participants from the Northern Sweden Health and Disease Study (NSHDS). Making the most of the EGM dataset, we examined participant’s inflammatory status through the inflammatory score (and a corresponding unsupervised alternative) in relation to cancer onset as a common mechanism in disease initiation and progression. These associations were investigated for each type of cancer (breast cancer and B-cell NHL separately), and after stratification by time-to-diagnosis (TtD).

## Results

### Study population

Key characteristics of the study population are reported in Table 1, participants were predominantly from Sweden (60.6%) with a mean age of 52.8 years (SD = 7.7). Participants from EPIC-Italy mostly women (74.3%) tended to report a lower education level, more physical activity, and have a higher alcohol consumption compared to participants from NSHDS. Our study population includes 90 breast cancer (54.4% from EPIC-Italy) and 248 B-cell NHL (33.8% from EPIC-Italy) prospective cases which were diagnosed from 2 to 15.5 years after inclusion.

**Table 1:** Comparison of baseline characteristics of EPIC-Italy and NSHDS.

		All individuals (n = 663)		EPIC-Italy (n = 261)		NSHDS (n = 402)		P-values
		N	% or mean(sd)	N	% or mean(sd)	N	% or mean(sd)	
Gender	Male	244	36,8%	67	25,7%	177	44,0%	<0.001
	Female	419	63,2%	194	74,3%	225	56,0%	
Type	Case	338	51,0%	133	51,0%	205	51,0%	—
	Control	325	49,0%	128	49,0%	197	49,0%	
Disease	Breast cancer (cases)	167 (90)	25,2%	94 (49)	36,0%	73 (41)	18,2%	<0.001
	NHL (cases)	496 (248)	74,8%	167 (84)	64,0%	329 (164)	81,8%	
Age	Global	663	52.8(7.7)	261	53.5(8.1)	402	52.4(7.5)	0.430
BMI (kg/m2)	Global	663	26.0(3.9)	261	25.8(3.6)	402	26.2(4.1)	0.329
Phase	1	355	53,5%	194	74,3%	161	40,1%	<0.001
	2	308	46,5%	67	25,7%	241	59,9%	
Center	Central	116	17,5%	116	44,4%	0	0,0%	—
	North	104	15,7%	104	39,9%	0	0,0%	
	South	41	6,2%	41	15,7%	0	0,0%	
	Umea	402	60,6%	0	0,0%	402	100,0%	
Smoking status	Never	326	49,2%	127	48,7%	199	49,5%	0.807
	Former	183	27,6%	70	26,8%	113	28,1%	
	Current	154	23,2%	64	24,5%	90	22,4%	
Alcohol (g/day)	Global	663	7.1(12.5)	261	12.5(17.6)	402	3.6(5.0)	<0.001
Physical activity	Inactive	179	27,1%	70	26,8%	109	27,1%	0.007
	Moderately inactive	266	40,1%	106	40,6%	160	39,8%	
	Moderately active	166	25,0%	54	20,7%	112	27,9%	
	Active	52	7,8%	31	11,9%	21	5,2%	
Education	None/primary	270	40,7%	146	55,9%	124	30,8%	<0.001
	Professional/technical	132	19,9%	30	11,5%	102	25,4%	
	Secondary	147	22,2%	55	21,1%	92	22,9%	
	University/college	114	17,2%	30	11,5%	84	20,9%	
Inflammatory score	Global	663	7.0(5.9)	261	6.8(6.1)	402	7.0(5.7)	0.244
PC1	Global	663	−0.01(3.0)	261	−0.003(3.0)	402	−0.02(3.1)	0.807

P-values are estimated with Ӽ2 tests, Student T-tests or Wilcoxon rank sum tests.

### Pre-diagnostic inflammatory score and breast cancer

The breast cancer population includes a total of 167 women (56.3% from EPIC-Italy) with a slightly higher proportion of cases in both cohorts after excluding individuals with missing data (Supplementary Table S1). The median time to diagnosis was 5.84 years and showed limited heterogeneity in both cohorts (5.35 years in EPIC-Italy and 6.43 years in NSHDS). BC cases from both cohorts were more inactive compared to controls (p = 0.05 in EPIC-Italy and NSHDS). In NSHDS, BC cases had lower BMI, experienced fewer pregnancies whereas no significant differences between cases and controls for any of the other baseline variables investigated were observed in EPIC-Italy (Supplementary Table S1). Women with BC from EPIC-Italy differed from those from NSHDS by age at menarche, parity, the use of hormone replace therapy and their menopausal status (Supplementary Table S2).

Our analyses based on the full BC population suggested a lower inflammatory score in BC cases compared to controls (model 1: β = −0.96, p = 0.333, Table 2A), but that difference did not reach statistical significance. Consistent estimates and similar conclusions can be drawn for the analyses stratified by cohort (model 1: β = −1.12, p = 0.436 and β = −0.64, p = 0.640 respectively for EPIC-Italy and NSHDS, Table 2A). Adjusting for either behavioural, socioeconomic, or hormonal factors, or in the fully adjusted models, effect size estimates showed consistent signs, and associations did not reach statistical significance (p > 0.09 across all models investigated, Table 2A, Supplementary Fig. S1). Stratification by time to diagnosis revealed statistically significant lower inflammatory score in BC cases diagnosed less than 6 years after enrolment (β = −2.88, p = 0.032, Table 2A). Results for each cohort separately showed consistent direction of association, but the lower inflammatory score in BC cases diagnosed less than 6 years after enrolment was borderline statistically significant in EPIC-Italy (β = −3.52, p = 0.057) and non-significant in NSHDS (β = −3.58, p = 0.196, Table 2A, Supplementary Fig. S1). No associations were observed in the pooled population or by cohort for BC cases diagnosed 6 years after enrolment (β = −0.06, p = 0.970, Table 2A).

**Table 2:** Association of pre-diagnostic inflammatory score (A) and the first PC (B) with breast cancer case/control.

	Full Breast Cancer population										TtD ≤ 6 years*		TtD > 6 years*	
	Model 1		Model 1 + Behaviours		Model 1 + SEP		Model 1 + Hormonal		Fully adjusted model		Fully adjusted model		Fully adjusted model	
	β (SE)	P-value	β (SE)	P-value	β (SE)	P-value	β (SE)	P-value	β (SE)	P-value	β (SE)	P-value	β (SE)	P-value
**(A) Inflammatory score**														
All	−0.96 (0.99)	0.333	−1.28 (1.01)	0.208	−1.27 (1.02)	0.215	−1.35(1.05)	0.201	−1.72(1.09)	0.118	**−2**.**88(1**.**32)**	**0**.**032**	−0.06(1.43)	0.970
EPIC-Italy	−1.12 (1.43)	0.436	−2.03 (1.47)	0.170	−1.45 (1.45)	0.318	−1.33(1.57)	0.400	−2.36(1.63)	0.151	−3.52(1.81)	0.057	1.03(2.67)	0.701
NSHDS	−0.64 (1.37)	0.640	−0.25 (1.54)	0.871	−0.86 (1.47)	0.558	−1.72(1.57)	0.278	−1.75 (1.88)	0.356	−3.58 (2.70)	0.196	−2.56 (1.88)	0.181
**(B) Principal component 1**														
All	−0.56 (0.53)	0.297	−0.74 (0.54)	0.175	−0.64 (0.55)	0.241	−0.87(0.55)	0.166	−1.00(0.57)	0.083	**−1**.**55(0**.**70)**	**0**.**029**	−0.09(0.75)	0.906
EPIC-Italy	−0.75 (0.77)	0.335	−1.25 (0.81)	0.123	−0.87 (0.79)	0.273	−0.83(0.84)	0.322	−1.30(0.88)	0.147	−1.90(1.04)	0.074	0.83(1.42)	0.564
NSHDS	−0.17 (0.71)	0.810	−0.004(0.77)	0.996	−0.23 (0.76)	0.760	−0.93 (0.81)	0.253	−0.85 (0.92)	0.361	−1.45 (1.22)	0.242	−1.29 (1.02)	0.214

Pooled cohorts (n = 167), EPIC-Italy (n = 94) and NSHDS (n = 73).

Model 1 is adjusted for age and cohort in the overall BC population; for age and center in EPIC-Italy and age in NSHDS.

β coefficient regression estimates inflammatory score’s (A) or PC1’s (B) difference in cases compared to controls.

*Additional analyses stratified by time from blood collection to cases’ diagnosis included for ‘less than 6 years’ strata 49 BC cancer cases: 32 in EPIC-Italy and 17 in NSHDS; for ‘higher than 6 years’ strata 41 BC cancer cases: 17 in EPIC-Italy and 24 in NSHDS.

Analyses using the first principal component (PC1, explaining 32.6% of the variance) as an alternative inflammatory score showed that our conclusions were robust to our definition of the inflammatory score and we identified the same significant association indicating a lower inflammatory score in the BC cases diagnosed less than 6 years after enrolment (β = −1.55, p = 0.029, Table 2B, Supplementary Fig. S2).

### Pre-diagnostic inflammatory score and B-cell NHL

Key characteristics of the B-cell NHL study population are given in Supplementary Table S3. Of the 248 β-cell malignancies cases diagnosed during follow-up, 84 arose from the Italian population and 164 from Sweden. The median time to diagnosis was 6.09 years (5.53 in EPIC-Italy and 6.38 in NSHDS). Cases and controls were on average 53.1 years old (Supplementary Table S3).

Our analyses of all B-cell NHL cases (i.e. pooling all histological subtypes) indicated a lower inflammatory score compared to controls (model 1: β = −1.28, p = 0.012 Table 3A). Consistent effect size estimates were observed in both cohorts separately, but the lower inflammatory score in B-cell NHL cases was only found statistically significant in the Swedish population (model 1: β = −0.89, p = 0.310 and β = −1.48, p = 0.019 for EPIC-Italy and NSHDS, respectively, Table 3A). The adjustment for socioeconomic variables, as for behaviours, lightly weakened these associations, but in the fully adjusted model, the lower inflammatory score in B-cell NHL cases was still significant in the pooled population (Table 3A – Supplementary Fig. S3).

**Table 3:** Association of pre-diagnostic inflammatory score (A) and the first PC (B) with B-cell non-Hodgkin lymphoma case/control.

	Full Lymphoma population								TtD ≤ 6 years*		TtD > 6 years*	
	Model 1		Model 1 + Behaviours		Model 1 + SEP		Fully adjusted model		Fully adjusted model		Fully adjusted model	
	β (SE)	P-value	β (SE)	P-value	β (SE)	P-value	β (SE)	P-value	β (SE)	P-value	β (SE)	P-value
**(A) Inflammatory score**												
All	**−1**.**28 (0**.**51)**	**0**.**012**	**−1**.**19 (0**.**51)**	**0**.**021**	**−1**.**21 (0**.**51)**	**0**.**019**	**−1**.**11 (0**.**52)**	**0**.**032**	−1.27 (0.65)	0.051	−1.06 (0.66)	0.108
EPIC-Italy	−0.89 (0.87)	0.310	−0.93 (0.89)	0.299	−0.96 (0.88)	0.277	−1.02 (0.90)	0.258	−1.87 (1.08)	0.087	0.05 (1.20)	0.965
NSHDS	**−1**.**48 (0**.**63)**	**0**.**019**	**−1**.**31 (0**.**64)**	**0**.**040**	**−1**.**36 (0**.**63)**	**0**.**031**	−1.19 (0.64)	0.062	−0.71 (0.82)	0.389	**−1**.**61 (0**.**79)**	**0**.**043**
**(B) Principal component 1**												
All	**−0**.**77 (0**.**26)**	**0**.**004**	**−0**.**71 (0**.**26)**	**0**.**007**	**−0**.**71 (0**.**26)**	**0**.**007**	**−0**.**65 (0**.**26)**	**0**.**013**	**−0**.**76 (0**.**32)**	**0**.**018**	−0.60 (0.33)	0.073
EPIC-Italy	−0.59 (0.40)	0.138	−0.66 (0.40)	0.101	−0.62 (0.40)	0.126	−0.70 (0.40)	0.083	**−1**.**14 (0**.**47)**	**0**.**017**	−0.13 (0.54)	0.816
NSHDS	**−0**.**85 (0**.**34)**	**0**.**012**	**−0**.**73 (0**.**34)**	**0**.**032**	**−0**.**76 (0**.**34)**	**0**.**024**	−0.64 (0.34)	0.059	−0.48 (0.43)	0.263	−0.82 (0.42)	0.053

Pooled cohorts (n = 496), EPIC-Italy (n = 167) and NSHDS (n = 329)

Model 1 is adjusted for age, gender, phase and cohort in the overall all B-cell NHL population; for age, gender, phase and center in EPIC-Italy and age, gender, phase in NSHDS. β coefficient regression estimates inflammatory score’s (A) or PC1’s (B) difference in cases compared to controls.

*Additional analyses stratified by time from blood collection to cases’ diagnosis included for ‘less than 6 years’ strata 129 B-cell NHL cancer cases: 49 in EPIC-Italy and 80 in NSHDS; for ‘higher than 6 years’ strata 119 B-cell NHL cancer cases: 35 in EPIC-Italy and 84 in NSHDS.

After stratification on time to diagnosis, we observed a borderline significant lower inflammatory score in cases diagnosed less than 6 years after inclusion in the pooled B-cell NHL population (β = −1.27, p = 0.051, Table 3A). A consistent estimate of the effect size was observed in cases diagnosed more than six years after enrollment, without the difference reaching statistical significance (β = −1.06, p = 0.108). B-cell NHL cases diagnoses more than 6 years after enrolment showed significantly lower inflammatory score only in the NSHDS samples. As before, results using PC1 as an alternative inflammatory score suggested highly consistent results (Table 3B, Supplementary Fig. S4).

## Discussion

Our analyses identified a general lower burden of inflammation in prospective BC and B-cell NHL cancer cases. Although non-significant, BC cases had a lower inflammatory score compared to controls in the pooled population and in both cohort. This difference was mainly driven by BC cases diagnosed less than 6 years after enrolment, for whom the inflammatory score was significantly lower than in controls. No significant differences were observed after stratification by BC histological subtypes in both oestrogen receptor positive and negative BC (Supplementary Table S4).

B cell NHL cases also had a significantly lower inflammatory score compared to controls in the pooled population and in NSHDS. Additional analyses showed a lower inflammatory score in all histological subtype cases, statistically significant in the largest subtype group of multiple myeloma (27.4% of B-cell NHL cases, Supplementary Table S5). Our results were robust to the definition of the inflammatory score and were not affected by adjusting for the main potential confounders.

Several limitations of this study should be considered. Our study population remains limited in size especially the stratified analyses by cohort, by histological subtypes and time to diagnoses. Our results showed overall similar trends but reduced strength of associations, which might indicate limited statistical power. The two cohorts we used may be heterogeneous in terms of population genetics, exposure profiles, lifestyle factors and study design-related source of variability, explaining some result differences between the two cohorts. Only BC and B-cell NHL cases were included in the EGM project, hence limiting our ability to investigate the role of inflammation in various cancer types. Additionally, a minimum of two years elapsed between enrolment and diagnosis. Since cancer development is likely to have a long latency period, over many years, we cannot rule out the presence of abnormal cells and cancer cases may already have been in a preclinical state at enrolment. Furthermore, we measured the inflammatory proteins at a single time point which may not reflect the long term inflammatory status of an individual and does not allow us to consider the speed of cancer progression in relation to longitudinal inflammatory burden. Finally, we cannot rule out the possibility that other factors may contribute to the association between inflammation and cancer risk. Furthermore variables relating to the inflammatory system, such as infection status and/or medication could not be accounted for.

Our study has a number of strengths. First, the prospective design limits both recall and reverse causation bias which can be induced by the disease itself, treatments or lifestyle changes after diagnosis. We also had access to a wide range of information about lifestyle factors and hormonal status which allowed us to adjust for important potential confounders. Additionally, the availability of two cohorts allowed for independent confirmation of the observed signals and comparison of the inflammatory score and cancer association results between two countries. Moreover, compared to most previous prospective studies, we measured and combined into a score a large panel of inflammatory proteins to capture the inflammatory load. We know of two studies which have examined several markers of inflammation both individually and jointly as an inflammatory score and have reported that the use of a score is a robust method to explore the association between inflammatory load and future risk of cancer^27,29^. Allin *et al*. reported a positive association between an inflammatory score derived from three inflammatory biomarkers (CRP, fibrinogen and whole blood leukocyte count) and future risk of colorectal, lung and BC during a median follow-up period of 4.8 years in a Danish general population^27^. To test the robustness of the definition of the inflammatory score, we defined, as an unsupervised alternative, a score using the first principal component obtained from the 28 inflammatory related proteins. Our results and conclusions remained stable with either score.

As a general pattern, behavioural factors and education did not seem to explain a substantial part of case-control differences in the inflammatory score. An unhealthy lifestyle, including smoking^30^, heavy drinking^31^, physical inactivity^32^ and body mass index is related to higher chronic inflammation and adverse health outcomes^33^. In our study, after controlling for behavioural and education, no significant changes were observed in the associations indicating that these variables do not act as confounder in the observed association between inflammation and cancer, suggesting that inflammation may be causally linked to cancer risk.

Our study remains however restricted to a limited set of potential confounders and mediators, hence hampering the generalisability of our conclusion.

A vast majority of the studies looking at the association between inflammation and BC risk used the CRP and a recent meta-analysis concludes a modest statistically significant positive association between CRP concentration and breast cancer risk^11,34^. Our results may appear to be contradictory. This may be due to our approach which summarises the association of 28 inflammatory related proteins with disease risk using a score to better capture the inflammatory status and improve statistical power but also attributable to the timing in which blood samples were collected and cancer diagnosed, or to other factors or confounders. Several epidemiological studies have explored the link between circulating marker of adiposity and inflammation in relation to BC risk with the underlying idea that obesity associated inflammation may increase the risk of cancer^9,34–36^. The release of inflammatory mediators may then take place in the adipocytes and might not be apparent at the same level in the circulatory system^36,37^. The time from blood collection to cancer diagnosis may change according to studies and influence results. In our results a lower inflammatory score in BC cases compared to controls was only observed in those diagnosed less than 6 years after enrolment. Early BC cases may present inflammatory differences compared to those developing BC cancer later or may be already in a pre-disease stage caracterized by a lower inflammatory score suggesting that inflammatory status differs according to BC cancer evolution time.

Numerous studies have investigated the association between pre-diagnostic inflammation and NHL using cytokines, chemokines and other immune markers independently. These studies showed a deregulation of these biomarkers with future risk of NHL suggesting subclinical dysfunction as a consistent risk factor^16–21,24,38^. In a previous study in a subset of EPIC-Italy’s population, the authors reported association between lower levels of IL-2, INF-y and upper levels of ICAM and risk of NHL^18^. In another study, it has been shown that lower levels of leptin was associated with increased risk of NHL and upper levels of IL-10 increase risk of B-NHL; they also found no association between a composite score from a principal component and future risk of NHL^20^. A case control study in the PLCO trial reported an increased risk of NHL with elevated serum levels of TNF-R1 and sCD27^17^. Our results demonstrate that future cases of NHL had a lower inflammatory score compared to controls. This provides further support for the subtle status of deregulated immune response or failure to modulate the immune response appropriately in relation to NHL risk. It is also established that NHL is one of the most common malignancies occurring after immunosuppressive therapy use due to transplantation^39,40^.

Inflammation has multifaceted role in cancer including initiation, promotion and progression. Deregulation of biological processes can lead towards chronic inflammation or play in immunosuppressive roles. Our results from a case-control study nested within 2 Europeans cohorts focusing on 2 specific cancers (BC and B-cell NHL) suggest an association between the presence of cancer and a lower inflammatory score and support the complex role of chronic inflammation in cancer development. The use of the PC1 aimed to measure the inflammation variability captured by the 28 proteins. These findings call for further studies to better understand how inflammatory changes evolve involving larger populations, larger variety of cancer types and repeated measures of larger panels of inflammatory markers.

## Methods

### Study population

The Envirogenomarkers study (EGM, www.envirogenomarkers.net) was designed as two nested case-control studies and includes participants from the Italian component of the European Prospective Investigation into Cancer and nutrition^41^ (EPIC-Italy) and the Northern Sweden Health and Disease Study^42^ (NSHDS), which have been described previously^43,44^. The Envirogenomarkers project and its associated studies, experimental protocols and methods were approved by the Regional Ethical Review Board of the Umeå Division of Medical Research, for the Swedish cohort, and the Florence Health Unit Local Ethical Committee, for the Italian cohort. All methods were carried out in accordance with the approved guidelines.

The EGM research project was to identify novel biomarkers associated with chronic diseases in which the environment might play an important role. EGM project was therefore focused on breast cancer and B-cell NHL. Exposure of environmental pollutants including polychlorinated biphenyls, polycyclic aromatic hydrocarbons, cadmium or lead were also collected.

EPIC-Italy includes 47,749 healthy participants aged 35–70 years old from 5 different areas: Turin, Varese, Florence, Naples, and Ragusa. Anthropometric measurement, lifestyle factors and blood samples were collected at recruitment (1993–1998). Standardized procedures were used to identify newly diagnosed cases of cancer based on automated linkages to cancer and mortality registries, municipal population offices and hospital discharge systems. In Naples follow-up information was collected through periodic personal contact. All participants signed a written informed consent and the ethical review boards of the International Agency for Research on Cancer, and of the collaborating institutions responsible for subject recruitment in each of the EPIC recruitment centers approved the study.

NSHDS is a prospective cohort study that included 95,000 participants from the general population from 1990 up to January 2008. It includes three sub-cohorts, of which the largest is the Västerbotten Intervention Programme (VIP) that mainly recruits individuals aged 40, 50 or 60 years. At initial recruitment, subjects were asked to complete a self-administered questionnaire to collect demographic, medical and lifestyle information and a separate self-administered food frequency questionnaire. Informed consent was obtained from all participants and a medical examination was conducted during which a blood sample was taken. Incident cancers occurring during follow-up were identified by linkage with the Swedish Cancer Registry and the local Northern Sweden Cancer Registry. NSHDS study was approved by the Local Ethical Committee in Umeå, Sweden. The sample selection strategy is described in Supplementary Fig. S5.

### Outcome

Our study population includes breast cancer (BC) and B-cell non-Hodgkin lymphoma (B-cell NHL) cases who were healthy at blood collection and were clinically diagnosed from 2 (minimum chosen duration to avoid the inclusion of cancer cases at enrolment) to 15.5 years after inclusion. Only invasive breast cancer cases were included in the study. All eligible B-cell NHL cases, including multiple myeloma were included. For each case, one suitable control was selected among participants in each cohort who were alive and free of cancer and were matched by center, gender, date of blood collection (+/−6 months), and age at recruitment (+/−2.5 years). We considered all B-cell NHL subtype together and in the text B-cell NHL includes multiple myeloma.

### Laboratory analyses

For each participant, a blood sample was collected at enrolment and stored in citrate (Italy) or EDTA (Sweden). Within two hours, plasma was separated and placed in cold storage. Biosamples underwent inflammatory profiling in two distinct phases and 32 of inflammation-related proteins were measured using the milliplex HCYTOMAG-60K and HSCYTMAG-60SK kits (Millipore, Billerca, MA), according to the manufacturer’s protocol^45^. Four analytes were excluded from further statistical analyses due to high rates of non-detection (>75%). Finally, 12 cytokines, 10 chemokines and 6 growth and angiogenic factors were measured for all participants (Supplementary Table S6). Protein levels below the level of detection were imputed based on a maximum likelihood estimation method exploiting the correlation structure across proteins to draw the missing values^46^. Levels of proteins were log-transformed to normalise their distributions. As previously proposed^47^, linear mixed models efficiently correct for technically-induced variation, which is potentially diluting the effects of interest. In practice, we adopted a two-step procedure first fitting a linear mixed model regressing protein levels (response) including a random intercept depending on technical covariates (microtiter plate). In a second step, we subtracted from the measured protein levels the random effect estimates measuring the variation linked to technical covariates to obtain denoised data (i.e. without the potential technical artefact)^48^.

### Inflammatory measure

As proposed previously^48^, and under the working hypothesis of a consistent contribution of each inflammatory marker to the overall inflammatory burden, we summarized the individual inflammatory status as a score derived from the 28 proteins assayed**:** for each protein, we defined a dichotomized indicator: “high concentration” = 1, and “low concentration” = 0 based on the highest quartile of the denoised protein concentrations, and summed across the 28 proteins these binary indicators^48^. Principal component analysis (PCA) is a mathematical algorithm that reduces the dimensionality of the data while retaining most of the variation in the data set^49^. PCA of these 28 inflammatory markers concentrations shows that 19 PCs explained more than 90% of the total variation seen in the dataset (Supplementary Fig. S6). As a continuous and hypothesis-free alternative score, we used the scores of the first principal component (PC1, explaining 32.6% of the total variance of the 28 proteins).

### Statistical analysis

We compared baseline characteristics of the total population and by cancer sub-population using the Chi-squared test or Fisher exact test for the categorical variables and T-test or Wilcoxon rank test for continuous variables. Categorical variables are reported as percentages while continuous variables are reported as mean (and standard deviation).

Our inflammatory score and its continuous alternative (PC1) were regressed against the disease outcome (case-control status and in BC and B-cell NHL population, separately) on the pooled population. We used the same model adjusting for potential confounders collected at enrolment for each type of cancer: age, gender, phase, cohort and center (Model 1). Potential confounders included behavioural factors: body mass index (BMI, continuous variable, kg/m2); smoking status (categorical: current, former, never); alcohol consumption (continuous, g/day); physical activity (categorical: inactive, moderately inactive, moderately active, active); and education (categorical: none/primary, professional/technical, secondary, university/college) as a proxy for socioeconomic status. Based on model 1, we controlled separately for behavioural factors (hereafter referred to as model 1 + behaviours) and for participant’s educational level (hereafter referred to as model 1 + socioeconomic position). The fully adjusted model included behavioural and socioeconomic factors listed above and was additionally adjusted for the reproductive/hormonal variables for BC analyses only. The latter comprised menopausal status (categorical: post-menopausal, pre-menopausal, unknown); contraceptive usage (categorical: yes, no); age at menarche (binary: ≤12 years old or >12 years old); menopausal hormone use (categorical: yes, no) and parity (quantitative discrete: 0, 1, 2, 3, >4).

### Sensitivity analyses

We fitted our models on data for each cohort separately (EPIC-Italy and NSHDS). To evaluate the impact of follow-up time and consider a potential effect of the preclinical phase of the disease, analyses were also stratified on the time to diagnosis as defined by the time elapsed between blood collection and clinical diagnosis: below or above the median time to diagnosis (6 years) across the entire population of cancer cases. We used the same time to diagnosis cut-off (below or above 6 years) for each type of cancer since the median time to diagnosis vary little across cancer type (5.84 years in BC and 6.09 years in B-cell NHL). In these analyses, cases were compared to all controls to preserve statistical power. Analyses were also performed by BC and B-cell NHL histological subtypes.

Statistical analyses were performed in R v3.2^50^ using the RStudio environment v.0.99.484. All p-values with p < 0.05 were considered statistically significant.

### Availability of data and materials

The datasets used and/or analysed during the current study are available from the corresponding author on reasonable request.

## Electronic supplementary material

Supplementary Information
