# Supplementary material for: Association between low-grade inflammation and Breast cancer and B-cell Myeloma and Non-Hodgkin Lymphoma: findings from two prospective cohorts
Source: Sci Rep. 2018 Jul 17;8:10805. doi: 10.1038/s41598-018-29041-1 (PMC6050323; doi:10.1038/s41598-018-29041-1)

Association between low-grade inflammation and Breast cancer and B-cell Myeloma and Non-Hodgkin Lymphoma: findings from two prospective cohorts

Eloise Berger[1], Cyrille Delpierre[1], Fatemeh Saberi Hosnijeh[2,3], Michelle Kelly-Irving[1], Lutzen Portengen[2], Ingvar A. Bergdahl[4], Ann-Sofie Johansson[5], Vittorio Krogh[6], Domenico Palli[7], Salvatore Panico[8], Carlotta Sacerdote[9], Rosario Tumino[10], Soterios A. Kyrtopoulos[11], Paolo Vineis [12,13,14], Marc Chadeau-Hyam[2,13,14], Roel Vermeulen[2,13,*], Raphaële Castagné[1,13,14,*,¤] on behalf of the EnviroGenoMarkers project consortium

Additional file contains 6 Tables and 6 Figures

Supplementary Table S1: Baseline characteristics of Breast Cancer cases and matched controls within pooled cohorts, EPIC-Italy and NSHDS.

		Breast Cancer cases and controls								
		**All (n=167)**			**EPIC-Italy (n=94)**			**NSHDS (n=73)**		
		Cases (n=90) mean (sd) or N(%)	Controls (n=77) mean (sd) or N(%)	P-value	Cases (n=49) mean (sd) or N(%)	Controls (n=45) mean (sd) or N(%)	P-value	Cases (n=41) mean (sd) or N(%)	Controls (n=32) mean (sd) or N(%)	P-value
Age		51.5(7.0)	51.8(6.8)	0.726	50.5(7.3)	51.1(7.3)	0.723	52.7(6.4)	52.8(5.9)	0.927
Gender	Female	90(100%)	77(100%)	1	49(100%)	45(100%)	1	41(100%)	32(100%)	1
	Male	0	0		0	0		0	0	
Cohort	Italy	49(54.4%)	45(58.4%)	0.717	-	-	-	-	-	-
	Sweden	41(45.6%)	32(41.6%)		-	-		-	-	
Phase	1	90(100%)	77(100%)	1	49(100%)	45(100%)	1	41(100%)	32(100%)	1
	2	0	0		0	0		0	0	
Oestrogen receptor	Positive	45(50%)	0	-	24(49.0%)	0	-	21(51.2%)	0	-
	Negative	45(50%)	0		25(51.0%)	0		20(48.8%)	0	
Time to Diagnosis (years)		5.84 (2.49)	-	-	5.35 (2.50)	-	-	6.43 (2.38)	-	-
	<= 6	49(54.4%)	77(100%)		32(65.3%)	45(100%)		17(41.46%)	32(100%)	
	> 6	41(45.6%)	77(100%)		17(34.7%)	45(100%)		24 (58.54%)	32(100%)	
Center	North	14(15.6%)	14(18.2%)	0.696	14(28.6%)	14(31.1%)	0.610	-	-	1
	Central	27(30.0%)	27(35.1%)		27(55.1%)	27(60.0%)		-	-	
	South	8(8.9%)	4(5.2%)		8(16.3%)	4(8.9)		-	-	
	Umea	41(45.6%)	32(41.6%)		-	-		41(100%)	32(100%)	
BMI (kg/m^2)		24.5(3.2)	25.2(4.0)	0.278	24.5(3.3)	24.6(3.6)	0.858	24.4(3.0)	26.1(4.5)	0.086
Alcohol (g/day)		4.3(7.2)	5.2(9.3)	0.413	6.3(9.1)	7.6(11.3)	0.546	1.9(2.5)	2.0(3.2)	0.868
Education	None/primary	40(44.4%)	36(46.8%)	0.046	24(49.0%)	22(48.9%)	0.492	16(39.0%)	14(43.8%)	0.094
	Prof/technical	20(22.2%)	8(10.4%)		7(14.3%)	3(6.7%)		13(31.7%)	5(15.6%)	
	Secondary	13(14.4%)	22(28.6%)		11(22.5%)	15(33.3%)		2(4.9%)	7(21.9%)	
	Univ/college	17(18.9%)	11(14.3%)		7(14.3%)	5(11.1%)		10(24.4%)	6(18.8%)	
Physical activity	Inactive	33(36.7%)	16(20.8%)	0.104	17(34.7%)	7(15.6%)	0.052	16(39.0%)	9(28.1%)	0.051
	Mod. Inactive	32(35.6%)	40(52.0%)		21(42.9%)	25(55.6%)		11(26.8%)	15(46.9%)	
	Mod. Active	17(18.9%)	14(18.2%)		9(18.4%)	6(13.3%)		8(19.5%)	8(25.0%)	
	Active	8(8.9%)	7(9.1%)		2(4.1%)	7(15.6%)		6(14.6%)	0	
Smoking status	Current	25(27.8%)	25(32.5%)	0.748	17(34.7%)	19(42.2%)	0.555	8(19.5%)	6(18.8%)	0.970
	Former	17(18.9%)	12(15.6%)		9(18.4%)	5(11.1%)		8(19.5%)	7(21.9%)	
	Never	48(53.3%)	40(52.0%)		23(46.9%)	21(46.7%)		25(61.0%)	19(59.4%)	

Menopause	Premenopausal	33(36.7%)	32(41.6%)	0.796	32(65.3%)	28(62.2%)	0.831	1(2.4%)	4(12.5%)	0.304
	Postmenopausal	46(51.1%)	37(48.1%)		17(34.7%)	17(37.8%)		29(70.7%)	20(62.5%)	
	Unkown	11(12.2%)	8(10.4%)		0	0		11(26.8%)	8(25.0%)	
Contraceptives	No	41(45.6%)	43(55.8%)	0.242	23(46.9%)	27(60.0%)	0.289	18(43.9%)	16(50.0%)	0.778
	Yes	49(54.4%)	34(44.2%)		26(53.1%)	18(40.0%)		23(56.1%)	16(50.0%)	
Age at menarche	>12	72(80.0%)	64(83.1%)	0.751	34(69.4%)	35(77.8%)	0.493	38(92.7%)	29(90.6%)	1
	<12	18(20.0%)	13(16.9%)		15(30.6%)	10(22.2%)		3(7.3%)	3(9.4%)	
Parity	0	9(10.0%)	8(10.4%)	0.066	7(14.3%)	7(15.6%)	0.332	2(4.9%)	1(3.1%)	0.068
	1	22(24.4%)	15(19.5%)		11(22.5%)	12(26.7%)		11(26.8%)	3(9.4%)	
	2	31(34.4%)	29(37.7%)		17(34.7%)	18(40.0%)		14(34.2%)	11(34.4%)	
	3	21(23.3%)	9(11.7%)		12(24.5%)	4(8.9%)		9(22.0%)	5(15.6%)	
	>4	7(7.8%)	16(20.8%)		2(4.1%)	4(8.9%)		5(12.2%)	12(37.5%)	
Menopausal hormone use	No	67(74.4%)	60(77.9%)	0.732	40(81.6%)	40(88.9%)	0.486	27(65.9%)	20(62.5%)	0.960
	Yes	23(25.6%)	17(22.1%)		9(18.4%)	5(11.1%)		14(34.1%)	12(37.5%)	

P-values are estimated with χ^2 tests, Fisher exact tests, Student t tests or Wilcoxon rank sum tests.

Supplementary Table S2: Comparison of baseline characteristics of hormonal factors for BC population in EPIC-Italy and NSHDS. Participants with non-missing value for any hormonal factors were included in the analyses (N=167).

		BREAST CANCER				
		EPIC-Italy (n=94)		NSHDS (n=73)		
		N	%	N	%	P-values
**Menopause**	Premenopausal	60	63.8%	5	6.9%	**<0.001**
	Postmenopausal	34	36.2%	49	67.1%	
	Unkown	0	0	19	26.0%	
**Contraceptives**	No	50	53.2%	34	46.6%	**0.489**
	Yes	44	46.8%	39	53.4%	
**Age at menarche**	>12	69	73.4%	67	91.8%	**0.005**
	<12	25	26.6%	6	8.2%	
**Menopausal hormone use**	No	80	85.1%	47	64.4%	**0.003**
	Yes	14	14.9%	26	35.6%	
**Number of pregnancies**	0	9	9.6%	3	4.1%	**0.534**
	1	19	20.2%	14	19.2%	
	2	28	29.8%	25	34.2%	
	3	22	23.4%	14	19.2%	
	>4	16	17.0%	17	23.3%	
**Parity**	0	14	14.9%	3	4.1%	**0.007**
	1	23	24.5%	14	19.2%	
	2	35	37.2%	25	34.2%	
	3	16	17.0%	14	19.2%	
	>4	6	6.4%	17	23.3%	

P-values are estimated with χ^2 tests and Fisher exact tests.

Supplementary Figure S1: (a) Boxplot of Inflammatory score by all Breast Cancer case/control type and after stratification by time to diagnosis. (b) Multiple regression analyses for case/control status and pre-diagnostic inflammatory score in breast cancer population and by cohort. β coefficient regression estimates score's difference in cases compared to controls. Model 1 is adjusted for age and cohort/center. Additional analyses stratified by time from blood collection to cases' diagnosis included for 'less than 6 years' strata 49 BC cancer cases: 32 in EPIC-Italy and 17 in NSHDS; for 'higher than 6 years' strata 41 BC cancer cases: 17 in EPIC-Italy and 24 in NSHDS.

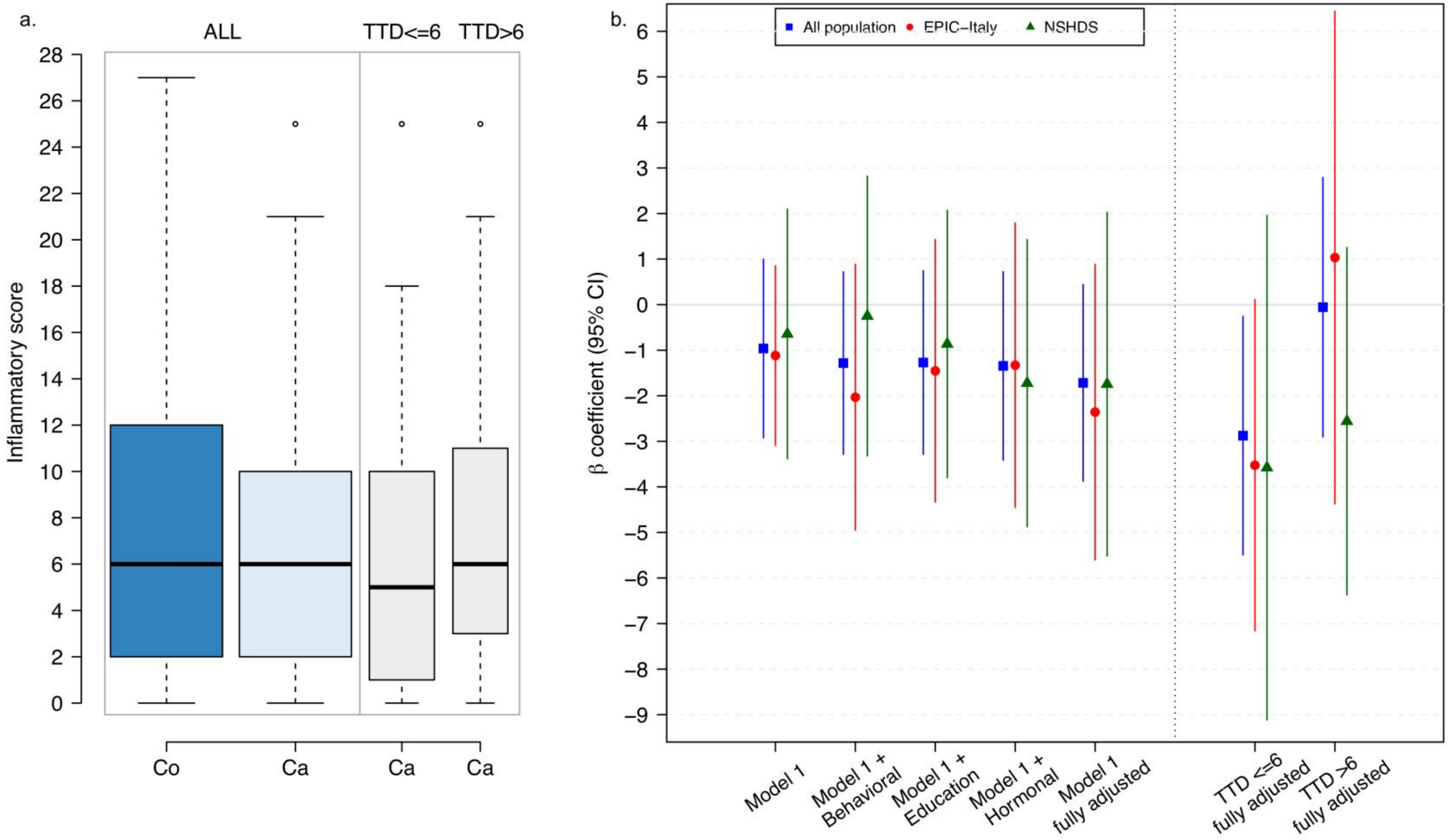

Supplementary Figure S2: (a) Boxplot of PC1 by all Breast Cancer case/control type and after stratification by time to diagnosis. (b) Multiple regression analyses for case/control status and PC1 in breast cancer population and by cohort. β coefficient regression estimates score's difference in cases compared to controls. Model 1 is adjusted for age and cohort/center. Additional analyses stratified by time from blood collection to cases' diagnosis included for 'less than 6 years' strata 49 BC cancer cases: 32 in EPIC-Italy and 17 in NSHDS; for 'higher than 6 years' strata 41 BC cancer cases: 17 in EPIC-Italy and 24 in NSHDS.

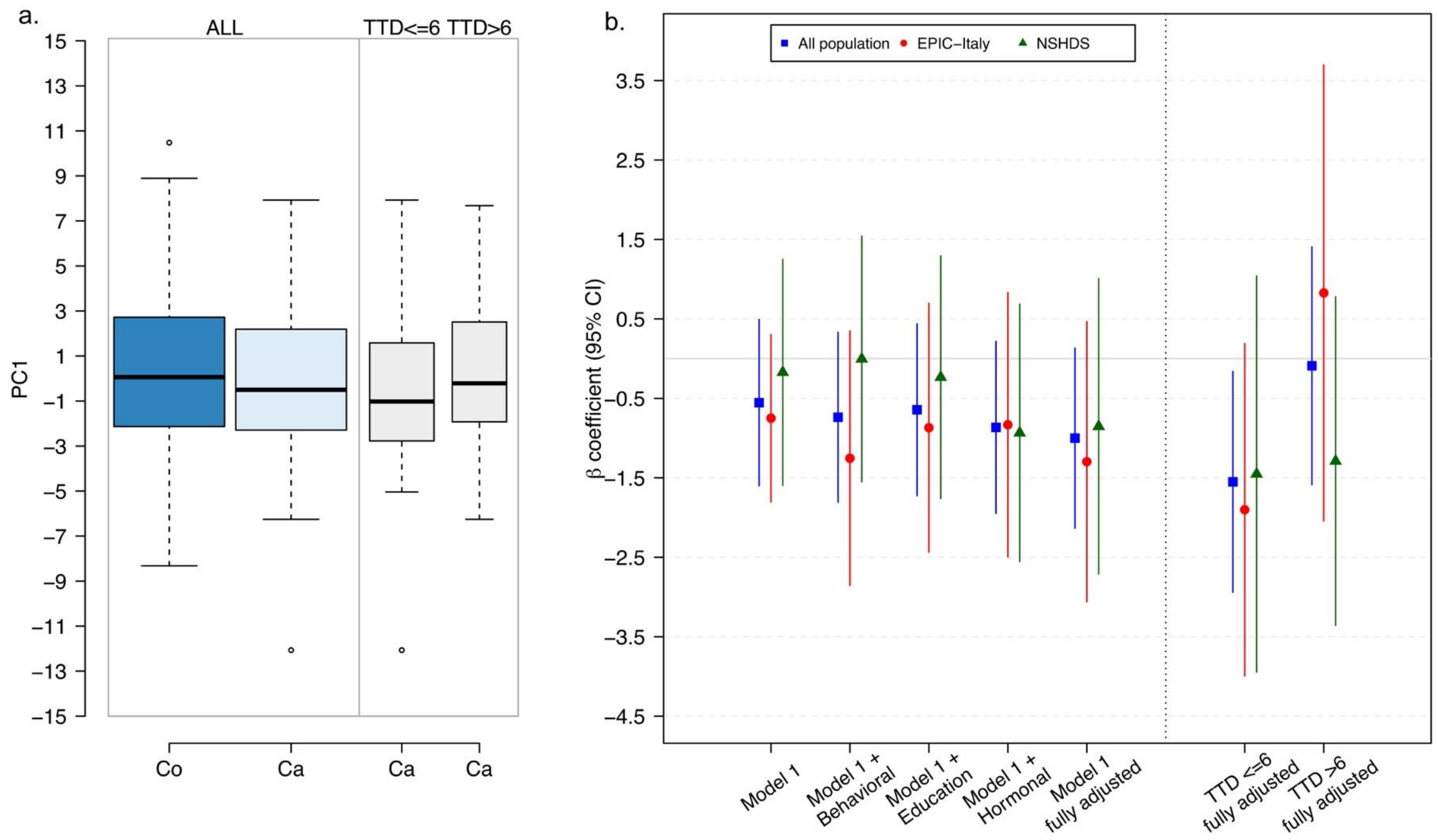

Supplementary Table S3: Baseline characteristics of B-cell non-Hodgkin lymphoma cases and matched controls within pooled cohorts, EPIC-Italy and NSHDS.

		B-cell Non-Hodgkin Lymphoma cases and controls								
		Pooled cohorts (n=496)			EPIC-Italy (n=167)			NSHDS (n=329)		
		Cases (n=248) mean (sd) or N(%)	Controls (n=248) mean (sd) or N(%)	P-values*	Cases (n=84) mean (sd) or N(%)	Controls (n=83) mean (sd) or N(%)	P-values*	Cases (n=164) mean (sd) or N(%)	Controls (n=165) mean (sd) or N(%)	P-values*
Age		53.1(7.88)	53.2(8.12)	0.492	55.0(8.1)	55.0(8.2)	0.967	52.2(7.6)	52.4(8.0)	0.323
Gender	Female	125(50.4%)	127(51.2%)	0.928	50(59.5%)	50(60.2%)	1	75(45.7%)	77(46.7%)	0.953
	Male	123(49.6%)	121(48.8%)		34(40.5%)	33(39.8%)		89(54.3%)	88(53.3%)	
Cohort	Italy	84(33.9%)	83(33.5%)	1	-	-	-	-	-	-
	Sweden	164(66.1%)	165(66.5%)		-	-		-	-	
Phase	1	94(37.9%)	94(37.9%)	1	50(59.5%)	50(60.2%)	1	44(26.8%)	44(26.7%)	1
	2	154(62.1%)	154(62.1%)		34(40.5%)	33(39.8%)		120(73.2%)	121(73.3%)	
NHL subtype	BCLL	40(16.2%)	0	-	11(13.1%)	0	-	29(17.7%)	0	-
	DLBL	41(16.5%)	0		11(13.1%)	0		30(18.3%)	0	
	FL	37(14.9%)	0		20(23.8%)	0		17(10.4%)	0	
	MM	68(27.4%)	0		21(25%)	0		47(28.7%)	0	
	Others	62(25.0%)	0		21 (25%)	0		41(25%)	0	
Time to Diagnosis (years)		6.09 (2.90)	-	-	5.53 (2.31)	-	-	6.38 (3.14)	-	-
	</= 6	129 (52.02%)	248 (100%)		49 (58.33%)	83 (100%)		80 (48.78%)	165 (100%)	
	> 6	119 (47.98%)	248 (100%)		35 (41.67%)	83 (100%)		84 (51.22%)	165 (100%)	
Center	North	38(15.3%)	38(15.3%)	0.998	38(45.2%)	38(45.8%)	1	-	-	1
	Central	31(12.5%)	31(12.5%)		31(36.9%)	31(37.3%)		-	-	
	South	15(6.1%)	14(5.7%)		15(17.9%)	14(16.9%)		-	-	
	Umea	164(66.1%)	165(66.5%)		-	-		164(100%)	165(100%)	
BMI (kg/m^2)		26.4(3.7)	26.5(4.2)	0.998	26.6(3.7)	26.4(3.3)	0.958	26.3(3.7)	26.5(4.5)	0.963
Alcohol (g/day)		7.6(12.9)	8.3(14.2)	0.204	15.0(18.6)	16.3(21.5)	0.957	3.7(5.6)	4.2(4.9)	0.062
Education	None/primary	95(38.3%)	99(39.9%)	0.304	50(59.5%)	50(60.2%)	0.776	45(27.4%)	49(29.7%)	0.405
	Prof/technical	58(23.4%)	46(18.6%)		12(14.3%)	8(9.7%)		46(28.1%)	38(23.0%)	
	Secondary	49(19.7%)	63(25.4%)		13(15.5%)	16(19.3%)		36(21.9%)	47(28.5%)	
	Univ/college	46(18.6%)	40(16.1%)		9(10.7%)	9(10.8%)		37(22.6%)	31(18.8%)	
Physical activity	Inactive	66(26.6%)	64(25.8%)	0.423	22(26.2%)	24(28.9%)	0.593	44(26.8%)	40(24.2%)	0.605
	Mod. Inactive	102(41.1%)	92(37.1%)		34(40.5%)	26(31.3%)		68(41.5%)	66(40.0%)	
	Mod. Active	66(26.6%)	69(27.8%)		19(22.6%)	20(24.1%)		47(28.6%)	49(29.7%)	
	Active	14(5.7%)	23(9.3%)		9(10.7%)	13(15.7%)		5(3.1%)	10(6.1%)	
Smoking status	Current	53(21.4%)	51(20.6%)	0.216	15(17.9%)	13(15.7%)	0.928	38(23.2%)	38(23.0%)	0.107
	Former	85(34.3%)	69(27.8%)		28(33.3%)	28(33.7%)		57(34.7%)	41(24.9%)	
	Never	110(44.3%)	128(51.6%)		41(48.8%)	42(50.6%)		69(42.1%)	86(52.1%)	

P-values are estimated with χ^2 tests, Student t tests or Wilcoxon ranks sum test.

Supplementary Figure 3: (a) Boxplot of Inflammatory score by all B-cell non-Hodgkin lymphoma case/control type and after stratification by time to diagnosis. (b) Multiple regression analyses for case/control status and pre-diagnostic inflammatory score in B-cell non-Hodgkin lymphoma population and by cohort. β coefficient regression estimates score's difference in cases compared to controls. Model 1 is adjusted for age, gender, phase and cohort/center. Additional analyses stratified by time from blood collection to cases' diagnosis included for 'less than 6 years' strata 129 B-cell NHL cancer cases: 49 in EPIC-Italy and 80 in NSHDS; for 'higher than 6 years' strata 119 B-cell NHL cancer cases: 35 in EPIC-Italy and 84 in NSHDS.

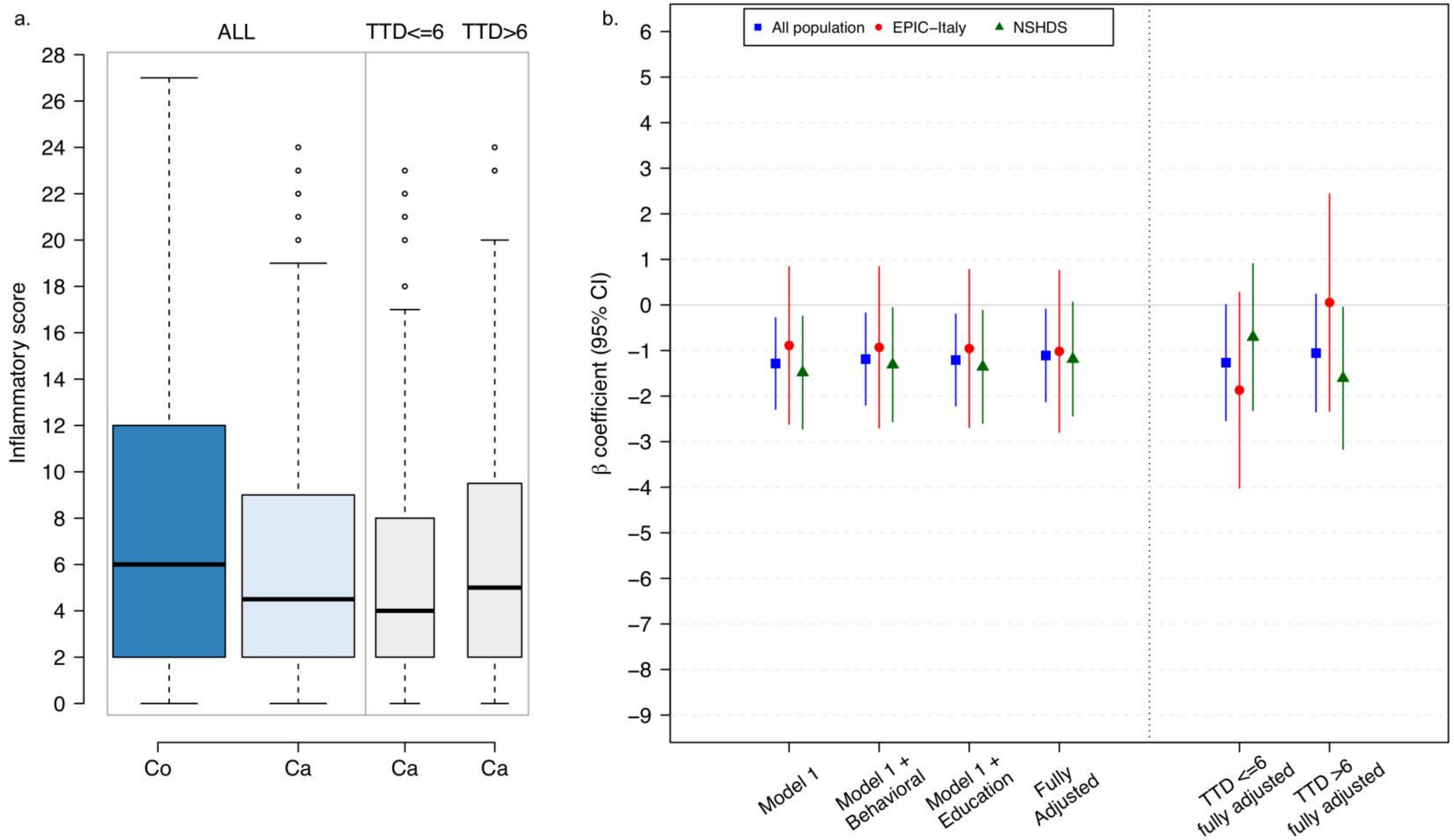

Supplementary Figure S4: (a) Boxplot of PC1 by all B-cell non-Hodgkin lymphoma case/control type and after stratification by time to diagnosis. (b) Multiple regression analyses for case/control status and PC1 in B-cell non-Hodgkin lymphoma population and by cohort. β coefficient regression estimates score's difference in cases compared to controls. Model 1 is adjusted for age, gender, phase and cohort/center. Additional analyses stratified by time from blood collection to cases' diagnosis included for 'less than 6 years' strata 129 B-cell NHL cancer cases: 49 in EPIC-Italy and 80 in NSHDS; for 'higher than 6 years' strata 119 B-cell NHL cancer cases: 35 in EPIC-Italy and 84 in NSHDS.

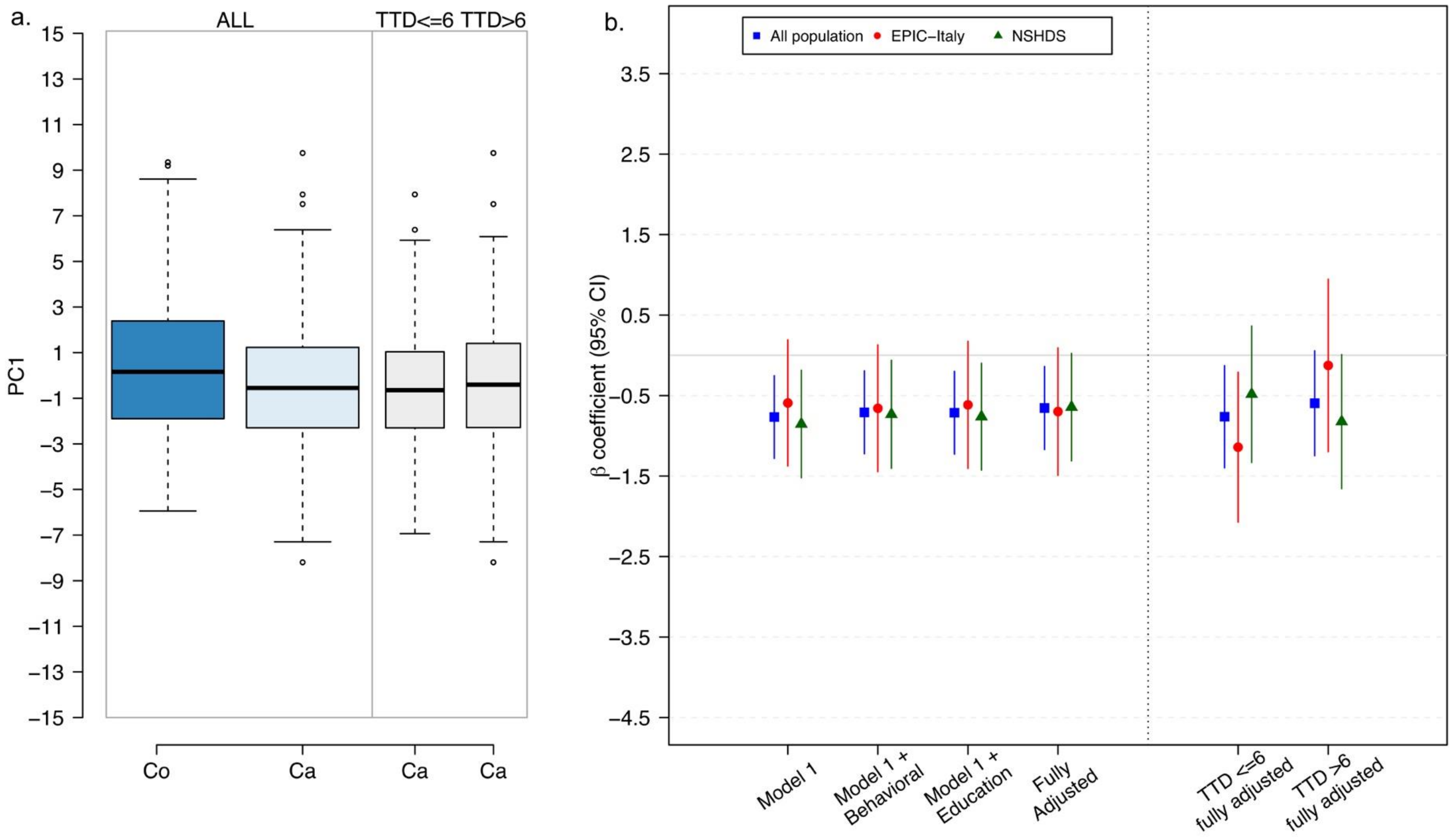

Supplementary Table S4: Association of pre-diagnostic inflammatory scores and breast cancer case/control after stratification by estrogen receptor

	ER +									
	**Model 1**		**Model 1 + Behaviours**		**Model 1 + SEP**		**Model 1 + Hormonal**		**Fully adjusted model**	
	β (SE)	P-value	β (SE)	P-value	β (SE)	P-value	β (SE)	P-value	β (SE)	P-value
**(A) Inflammatory score**										
All	-1.44 (1.13)	0.205	-1.45 (1.14)	0.207	-1.44 (1.16)	0.217	-1.70 (1.18)	0.151	-1.65 (1.21)	0.175
EPIC-Italy	-1.74 (1.64)	0.294	-2.82 (1.70)	0.104	-1.76 (1.68)	0.299	-1.46 (1.83)	0.429	-2.49 (2.00)	0.220
NSHDS	-0.91 (1.59)	0.570	0.60 (1.81)	0.743	-1.08 (1.71)	0.531	-2.54 (1.71)	0.146	-1.80 (2.13)	0.405
**(B) Principal component 1**										
All	-0.69 (0.61)	0.258	-0.69 (0.62)	0.270	-0.62 (0.62)	0.321	-0.91 (0.62)	0.142	-0.82 (0.64)	0.201
EPIC-Italy	-0.71 (0.89)	0.431	-1.19 (0.95)	0.216	-0.69 (0.91)	0.452	-0.48 (0.95)	0.616	-0.77 (1.06)	0.474
NSHDS	-0.39 (0.83)	0.638	0.26 (0.95)	0.783	-0.43 (0.90)	0.634	-1.35 (0.93)	0.155	-1.02 (1.17)	0.391

	ER -									
	**Model 1**		**Model 1 + Behaviours**		**Model 1 + SEP**		**Model 1 + Hormonal**		**Fully adjusted model**	
	β (SE)	P-value	β (SE)	P-value	β (SE)	P-value	β (SE)	P-value	β (SE)	P-value
**(A) Inflammatory score**										
All	-0.59 (1.28)	0.647	-1.05 (1.34)	0.435	-0.92 (1.34)	0.495	-1.49 (1.36)	0.276	-2.13 (1.43)	0.139
EPIC-Italy	-0.68 (1.83)	0.713	-1.25 (1.94)	0.522	-1.25 (1.90)	0.515	-1.02 (2.01)	0.614	-2.45 (2.13)	0.256
NSHDS	-0.63 (1.79)	0.726	-1.56 (2.01)	0.441	-0.68 (1.93)	0.726	-1.88 (2.19)	0.398	-3.02 (2.42)	0.221
**(B) Principal component 1**										
All	-0.49 (0.67)	0.468	-0.78 (0.71)	0.273	-0.61 (0.71)	0.389	-1.08 (0.69)	0.121	-1.39 (0.72)	0.057
EPIC-Italy	-0.80 (1.00)	0.425	-1.18 (1.08)	0.280	-1.06 (1.04)	0.315	-0.88 (1.05)	0.404	-1.74 (1.13)	0.131
NSHDS	-0.07 (0.88)	0.935	-0.65 (0.94)	0.494	-0.02 (0.94)	0.987	-0.87 (1.05)	0.412	-1.43 (1.07)	0.193

Supplementary Table S5: Association of pre-diagnostic inflammatory scores and B-cell non-Hodgkin lymphoma case/control after stratification by histologic subtype

	B-cell NHL - Follicular lymphoma							
	**Model 1**		**Model 1 + Behaviours**		**Model 1 + SEP**		**Fully adjusted model**	
	β (SE)	P-value	β (SE)	P-value	β (SE)	P-value	β (SE)	P-value
**(A) Inflammatory score**								
All	-0.77 (1.05)	0.465	-0.55 (1.07)	0.612	-0.60 (1.06)	0.571	-0.40 (1.08)	0.713
EPIC-Italy	-0.14 (1.48)	0.923	0.10 (1.52)	0.946	-0.19 (1.49)	0.897	0.14 (1.51)	0.928
NSHDS	-1.38 (1.50)	0.010	-0.94 (1.53)	0.537	-1.04 (1.51)	0.494	-0.62 (1.55)	0.687
**(B) Principal component 1**								
All	-0.27 (0.52)	0.609	-0.14 (0.53)	0.795	-0.16 (0.53)	0.758	-0.05 (0.53)	0.927
EPIC-Italy	-0.23 (0.67)	0.731	-0.04 (0.67)	0.954	-0.24 (0.67)	0.724	-0.02 (0.67)	0.978
NSHDS	-0.22 (0.79)	0.783	-0.02 (0.80)	0.982	0.04 (0.80)	0.963	0.23 (0.81)	0.775

	B-cell NHL - B-cell Chronic Lymphatic Leukemia							
	**Model 1**		**Model 1 + Behaviours**		**Model 1 + SEP**		**Fully adjusted model**	
	β (SE)	P-value	β (SE)	P-value	β (SE)	P-value	β (SE)	P-value
**(A) Inflammatory score**								
All	-1.03 (1.01)	0.311	-1.22 (1.02)	0.236	-1.22 (1.01)	0.231	-1.41 (1.03)	0.171
EPIC-Italy	-1.88 (1.97)	0.341	-2.10 (2.16)	0.333	-2.29 (1.99)	0.254	-2.58 (2.20)	0.244
NSHDS	-0.89 (1.18)	0.451	-0.74 (1.20)	0.539	-1.20 (1.18)	0.309	-0.99 (1.20)	0.411
**(B) Principal component 1**								
All	-0.17 (0.50)	0.738	-0.28 (0.50)	0.574	-0.29 (0.50)	0.557	-0.41 (0.50)	0.416
EPIC-Italy	-0.40 (0.87)	0.674	-0.54 (0.96)	0.576	-0.55 (0.89)	0.539	-0.71 (0.98)	0.471
NSHDS	-0.15 (0.61)	0.808	-0.12 (0.62)	0.844	-0.33 (0.61)	0.586	-0.27 (0.62)	0.664

	*B-cell NHL - Others**							
	**Model 1**		**Model 1 + Behaviours**		**Model 1 + SEP**		**Fully adjusted model**	
	β (SE)	P-value	β (SE)	P-value	β (SE)	P-value	β (SE)	P-value
**(A) Inflammatory score**								
All	-1.08 (0.84)	0.201	-1.10 (0.85)	0.198	-1.02 (0.84)	0.227	-1.02 (0.86)	0.236
EPIC-Italy	-0.07 (1.43)	0.961	-0.36 (1.56)	0.819	0.10 (1.44)	0.948	-0.23 (1.58)	0.885
NSHDS	-1.65 (1.04)	0.115	-1.51 (1.05)	0.153	-1.60 (1.03)	0.124	-1.45 (1.05)	0.169
**(B) Principal component 1**								
All	-0.57 (0.42)	0.169	-0.59 (0.42)	0.164	-0.52 (0.42)	0.215	-0.51 (0.42)	0.224
EPIC-Italy	-0.22 (0.62)	0.722	-0.46 (0.67)	0.496	-0.18 (0.63)	0.779	-0.45 (0.69)	0.514
NSHDS	-0.80 (0.55)	0.143	-0.80 (0.55)	0.147	-0.75 (0.54)	0.165	-0.75 (0.55)	0.172

	B-cell NHL - Diffuse large B-cell lymphoma							
	**Model 1**		**Model 1 + Behaviours**		**Model 1 + SEP**		**Fully adjusted model**	
	β (SE)	P-value	β (SE)	P-value	β (SE)	P-value	β (SE)	P-value
**(A) Inflammatory score**								
All	-0.87 (1.01)	0.393	-0.71 (1.04)	0.491	-0.62 (1.03)	0.543	-0.44 (1.05)	0.674
EPIC-Italy	0.21 (1.96)	0.915	-0.06 (2.05)	0.978	-0.17 (1.99)	0.931	-0.37 (2.08)	0.859
NSHDS	-0.81 (1.19)	0.496	-0.40 (1.23)	0.748	-0.36 (1.20)	0.766	0.06 (1.24)	0.963
**(B) Principal component 1**								
All	-0.61 (0.51)	0.235	-0.52 (0.52)	0.316	-0.42 (0.52)	0.414	-0.33 (0.53)	0.536
EPIC-Italy	-0.29 (0.85)	0.734	-0.44 (0.89)	0.623	-0.42 (0.87)	0.631	-0.58 (0.91)	0.523
NSHDS	-0.57 (0.64)	0.377	-0.34 (0.65)	0.600	-0.27 (0.64)	0.674	-0.07 (0.66)	0.921

	B-cell NHL - Multiple myeloma							
	**Model 1**		**Model 1 + Behaviours**		**Model 1 + SEP**		**Fully adjusted model**	
	β (SE)	P-value	β (SE)	P-value	β (SE)	P-value	β (SE)	P-value
**(A) Inflammatory score**								
All	-2.09 (0.80)	0.010	-1.88 (0.81)	0.021	-2.05 (0.81)	0.012	-1.83 (0.82)	0.026
EPIC-Italy	-2.36 (1.48)	0.113	-2.17 (1.52)	0.157	-2.34 (1.49)	0.120	-2.15 (1.55)	0.170
NSHDS	-2.14 (0.96)	0.027	-1.91 (0.96)	0.049	-2.03 (0.96)	0.035	-1.82 (0.96)	0.061
**(B) Principal component 1**								
All	-1.61 (0.41)	0.000	-1.48 (0.41)	0.000	-1.56 (0.41)	0.000	-1.43 (0.41)	0.001
EPIC-Italy	-1.48 (0.64)	0.023	-1.35 (0.66)	0.042	-1.49 (0.65)	0.024	-1.38 (0.67)	0.043
NSHDS	-1.72 (0.52)	0.001	-1.59 (0.52)	0.002	-1.64 (0.52)	0.002	-1.51 (0.52)	0.004

Supplementary Figure S5: Flow chart summarizing the participant selection of this study

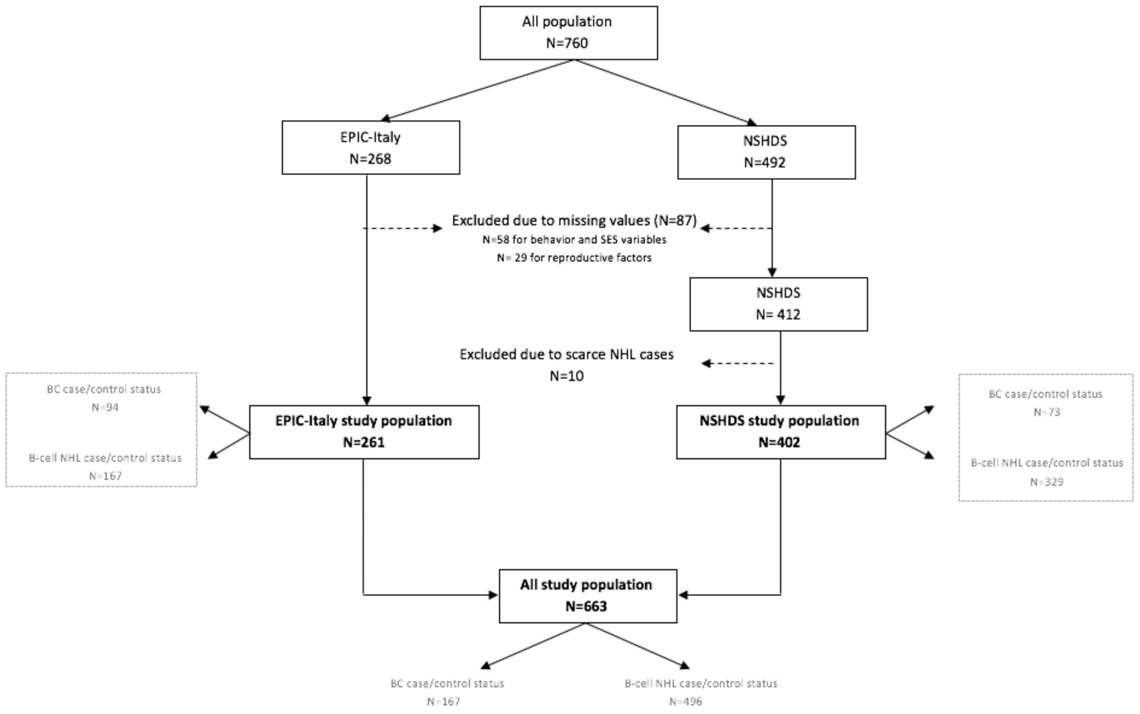

Scarce B-cell NHL cases includes Hodgkin's lymphomas (HL), T-cell Lymphoma (TNOS), 'unknown'.

Supplementary Table S6: Summary features of the 28 inflammatory markers used to determine inflammatory score

Manufacturer's name	Protein	Official symbol
Eotaxin	Chemokine (C-C motif) ligand 11	CCL11
Mcp-1	Monocyte chemotactic protein 1	CCL2
Mcp-3	Chemokine (C-C motif) ligand 7	CCL7
Mip-1a	Chemokine (C-C motif) ligand 3	CCL3
Mip-1b	Chemokine (C-C motif) ligand 4	CCL4
Mdc	Chemokine (C-C motif) ligand 22	CCL22
Fractalkine	Chemokine (C-X3-C motif) ligand 1	CX3CL1
Gro	Chemokine (C-X-C motif) ligand 1	CXCL1
Ip-10	CXCL10 chemokine (C-X-C motif) ligand 10	CXCL10
Il-8	Interleukin 8 //chemokine (C-X-C motif) ligand 8	IL8 // CXCL8
Infa 2	Interferon, alpha 2	IFNA2
Il-1b	Interleukin 1, beta	IL1B
Il-2	Interleukin 2	IL2
Il-4	Interleukin 4	IL4
Il-5	Interleukin 5	IL5
Il-6	Interleukin 6	IL6
Il-7	Interleukin 7	IL7
Il-10	Interleukin 10	IL10
Il-13	Interleukin 13	IL13
Infg	Interferon, gamma	IFNG
Scd401	Soluble CD40 ligand	CD40LG
Tnfa	Tumor necrosis factor	TNF
Egf	Epidermal growth factor	EGF
g-csf	Colony stimulating factor 3 (granulocyte)	CSF3
Gm-csf	Colony stimulating factor 2 (granulocyte-macrophage)	CSF2
Tgfa	Transforming growth factor, alpha	TGFA
Vegf	Vascular endothelial growth factor A	VEGFA
Fgf2	Fibroblast growth factor 2	FGF2

Supplementary Figure S6: Scree plot from the principal components analyses on the overall sample (N=760) for the 28 proteins (A). Contribution of the proteins level to the PC1, the red dashed line indicates the expected average contribution.

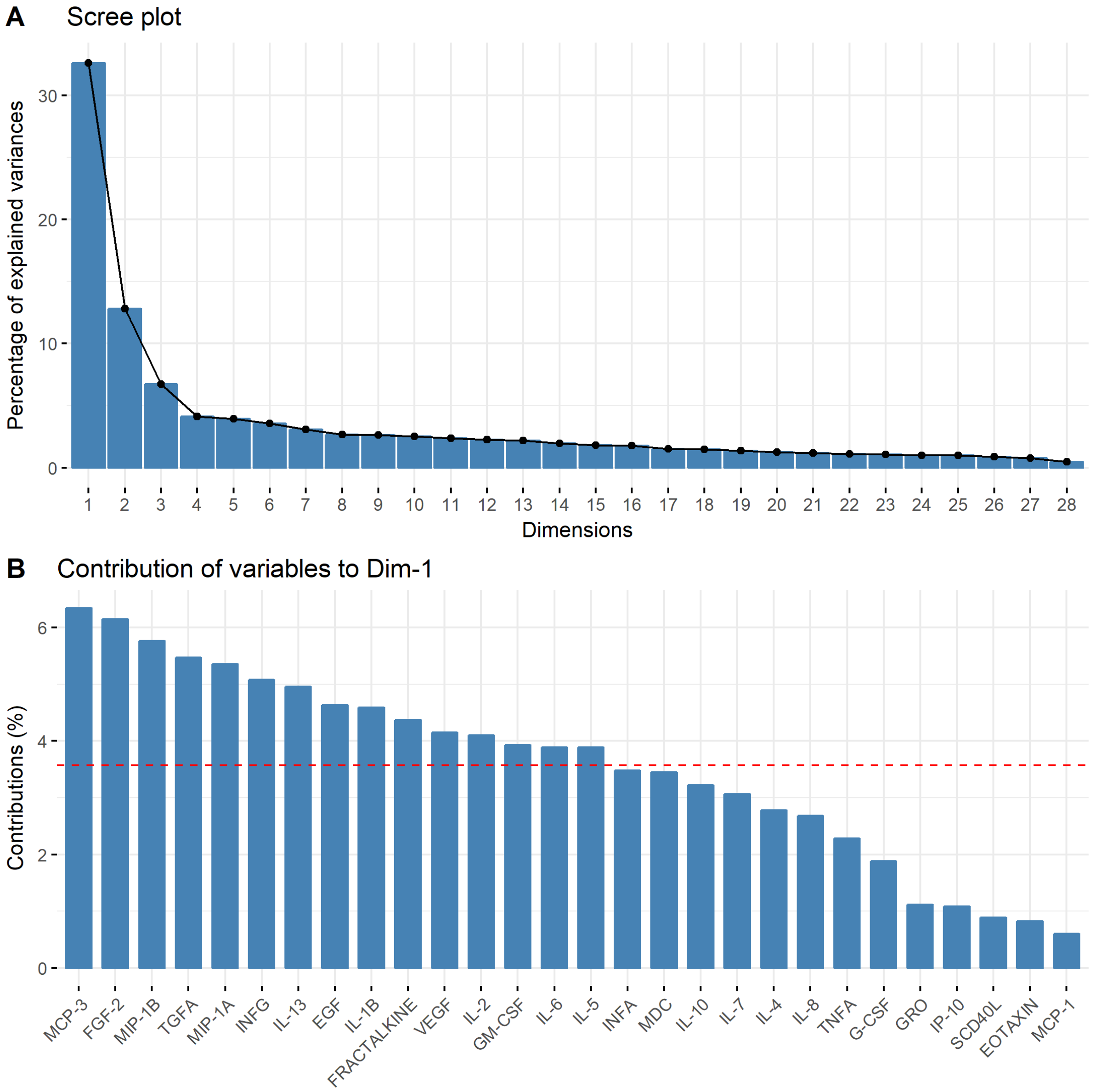

Supplement: Supplementary file 1 — Supplementary Information [file 41598_2018_29041_MOESM1_ESM.pdf]
